# Supplementary material for: mNSF: multi-sample non-negative spatial factorization
Source: Genome Biol. 2025 Jun 2;26:149. doi: 10.1186/s13059-025-03601-x (PMC12128389; doi:10.1186/s13059-025-03601-x)
Supplement: Supplementary file 1 — Additional file 1: Contains Figs. S1–S22 and Tables S1–S3 [file 13059_2025_3601_MOESM1_ESM.pdf]

SUPPLEMENTARY MATERIALS

mNSF: Multi-sample non-negative spatial factorization

Yi Wang, Kyla Woyshner, Chaichontat Sriworarat, Genevieve Stein-O'Brien, Loyal A Goff, Kasper D. Hansen

Contents

1 Supplemental Figures 2

2 Supplemental Tables 22

1 Supplemental Figures

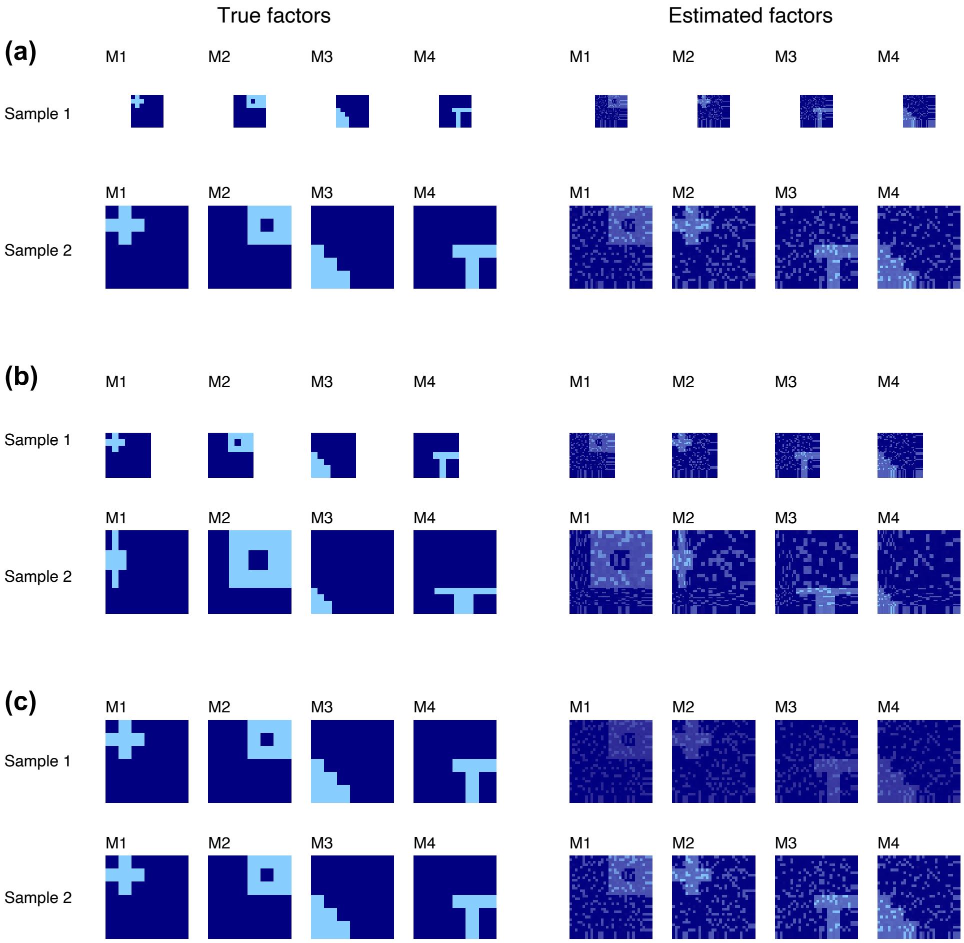

Fig S1. Multi-sample NSF performance on simulated data under additional scenarios. Like Figure 1 but under additional scenarios. True factors are in the first column, estimated factors in the second. **(a)** Size differences: samples with varying sizes of spatial patterns. **(b)** Distortion: the true factor is distorted between samples. (c) Sample-specific noise level.

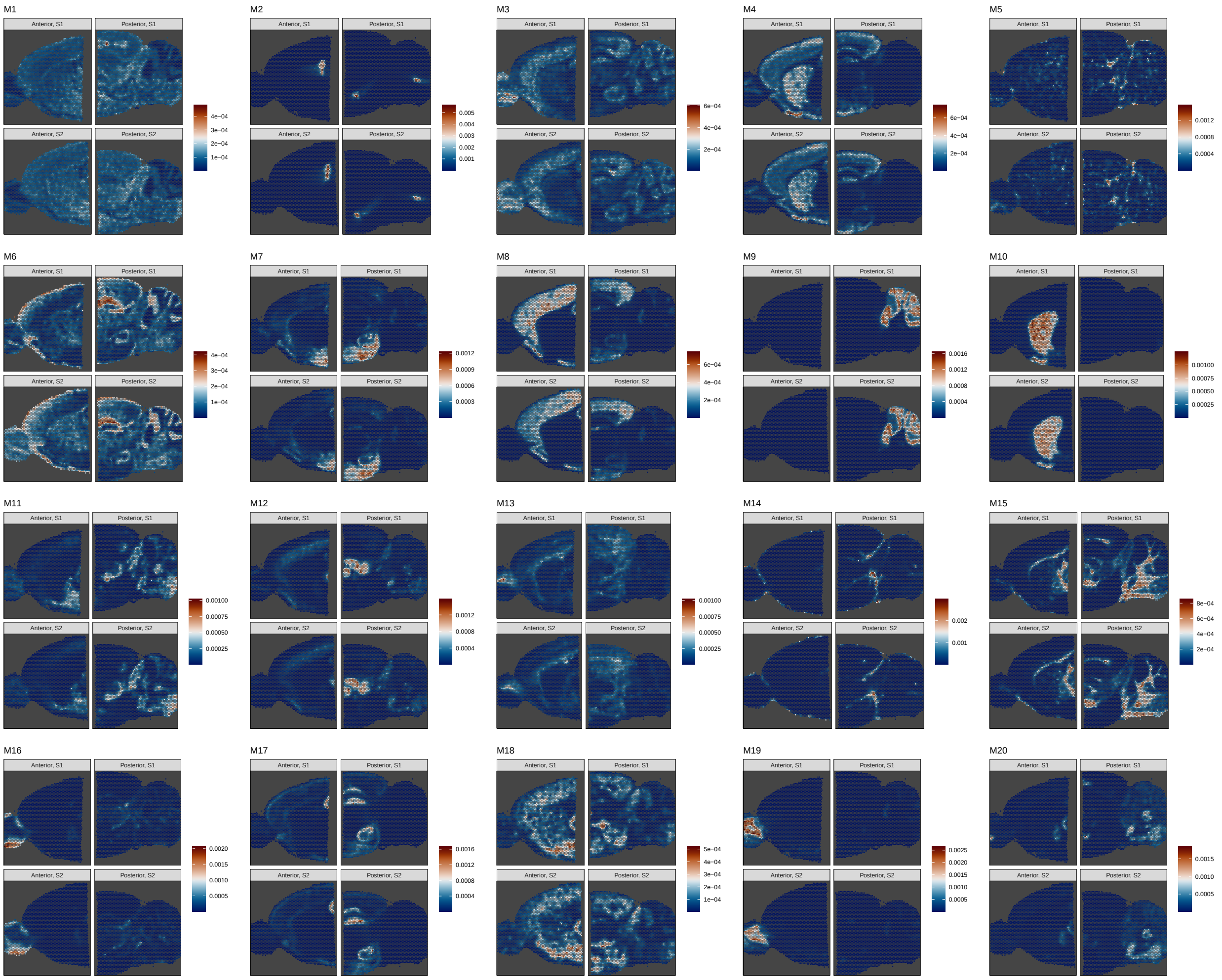

Fig S2. mNSF factors of mouse sagittal data show associations with the anatomical structure | The dataset is composed of four samples – two pairs of replicates, each for the anterior and the posterior region. Four-sample NSF is applied in this data, with twelve factors used. Each pair of replicates is in the same column in each subplot. Comparing the spatial pattern of each factor to a reference diagram of the mouse brain, it is easy to establish that factor 16 and 19 are enriched in olfactory bulb, and factor 9 is enriched in cerebellum.

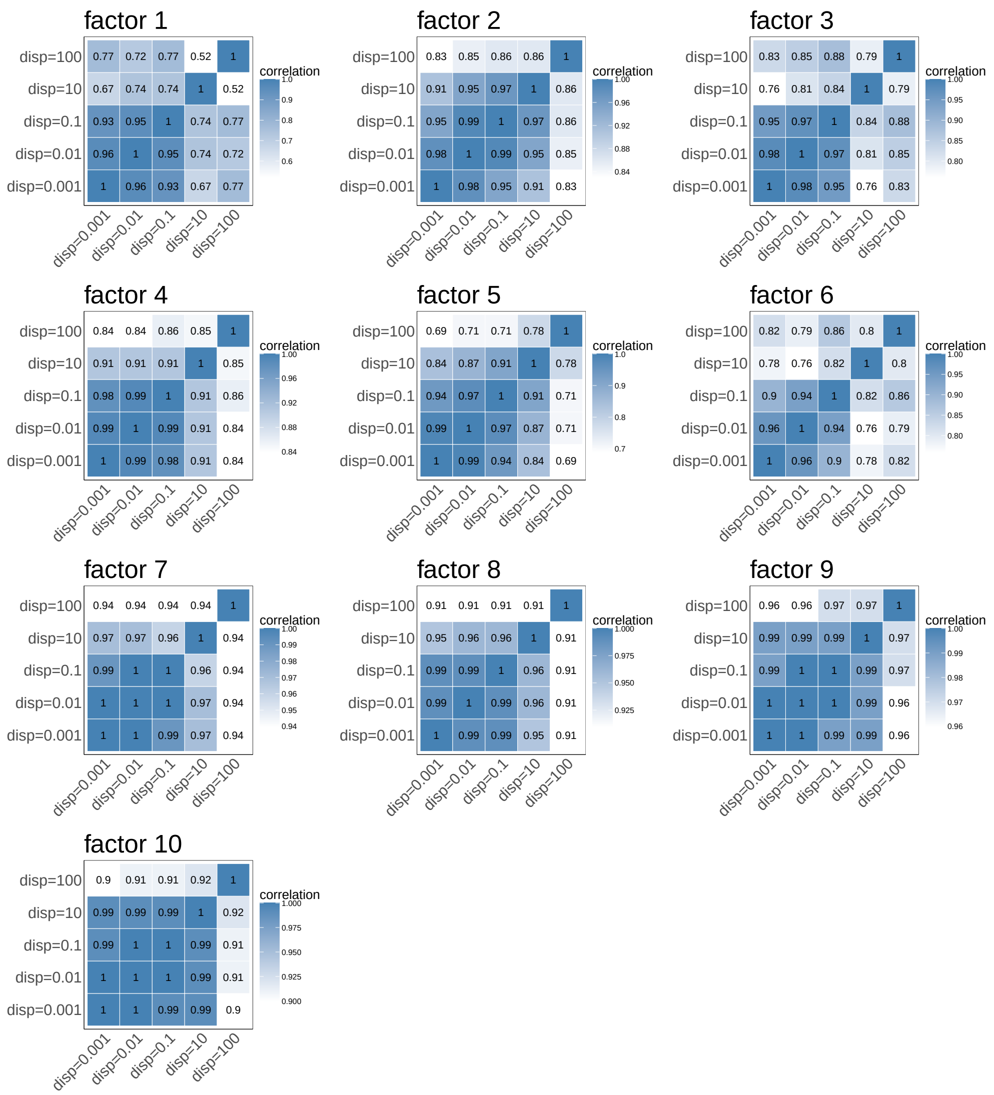

Fig S3. Sensitivity analysis of dispersion parameter on mouse sagittal brain data. Correlation matrices displaying the similarity between spatial factors obtained using different dispersion parameter values (ranging from 0.001 to 100) for factors 1-10 in the mouse sagittal brain dataset. Each heatmap represents one factor, with correlation values indicated by color intensity. The consistently high correlation values (generally > 0.8) across most dispersion parameter values demonstrate that mNSF produces stable results across a broad range of dispersion values (0.001, 0.01, 0.1, 10, and 100). Some factors show slightly lower correlations at dispersion=100.

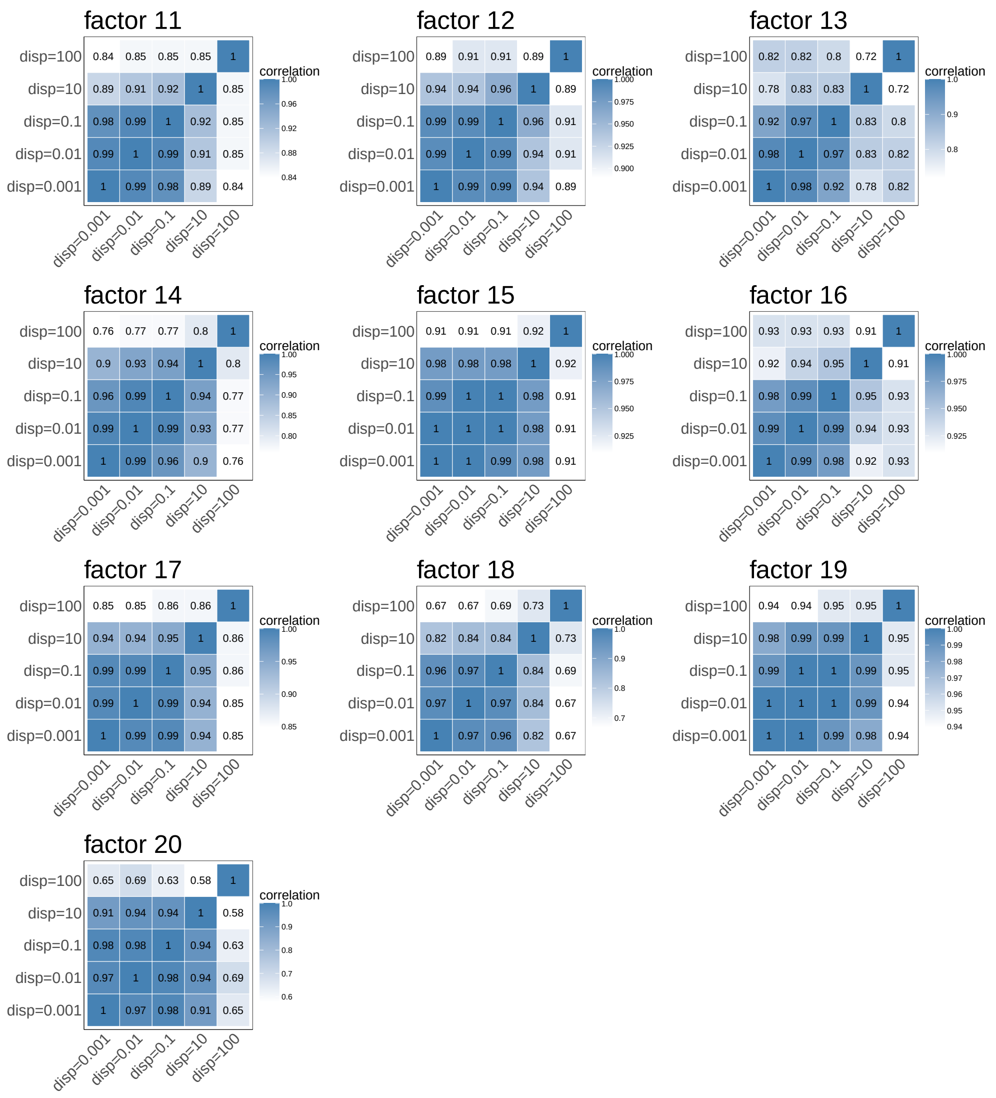

Fig S4. Sensitivity analysis of dispersion parameter on mouse sagittal brain data. Like Supplementary Figure S3 but for factors 11-20.

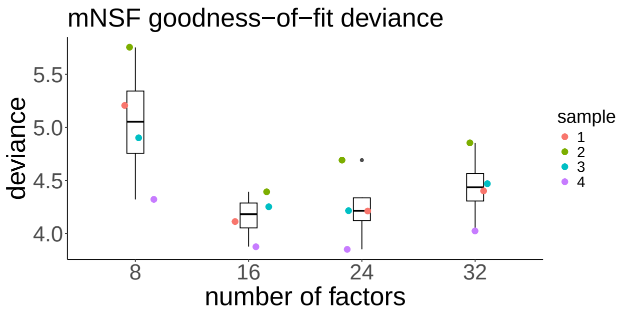

Fig S5. Evaluation of mNSF performance across different numbers of factors for mouse sagittal section data. Goodness-of-fit deviance for mouse sagittal section data.

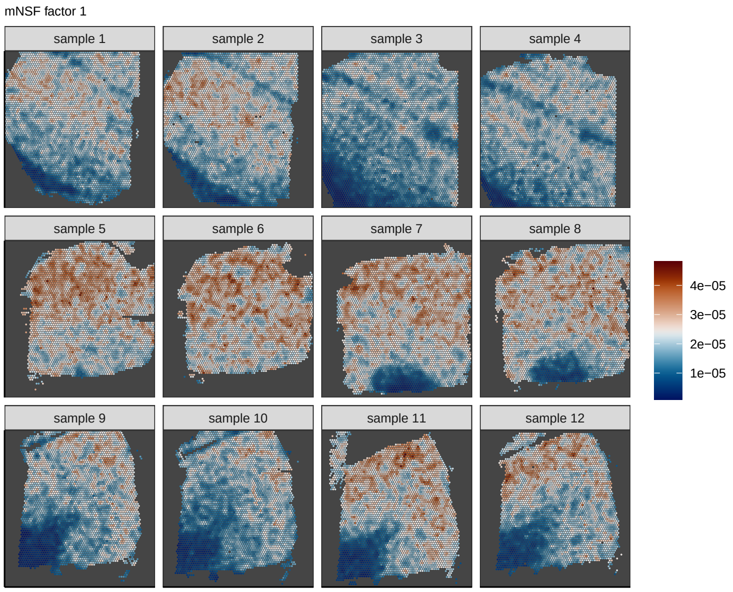

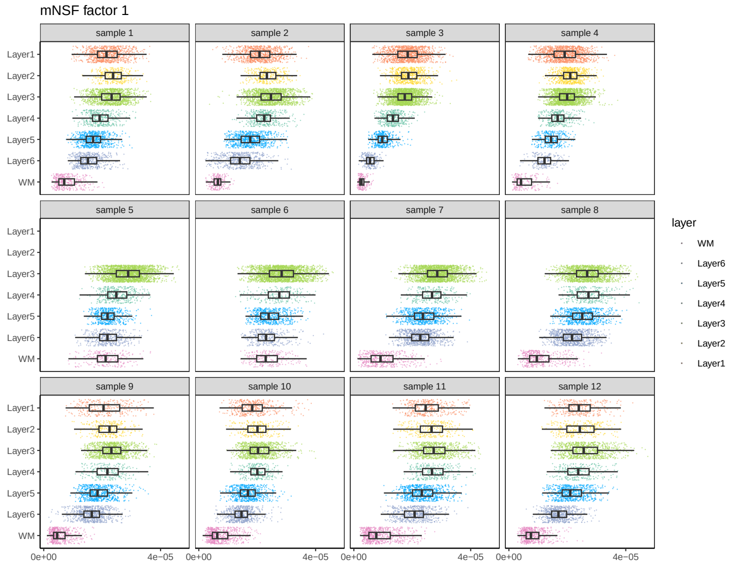

Fig S6. The value of each mNSF factor M1 for each of the 12 samples in DLPFC data

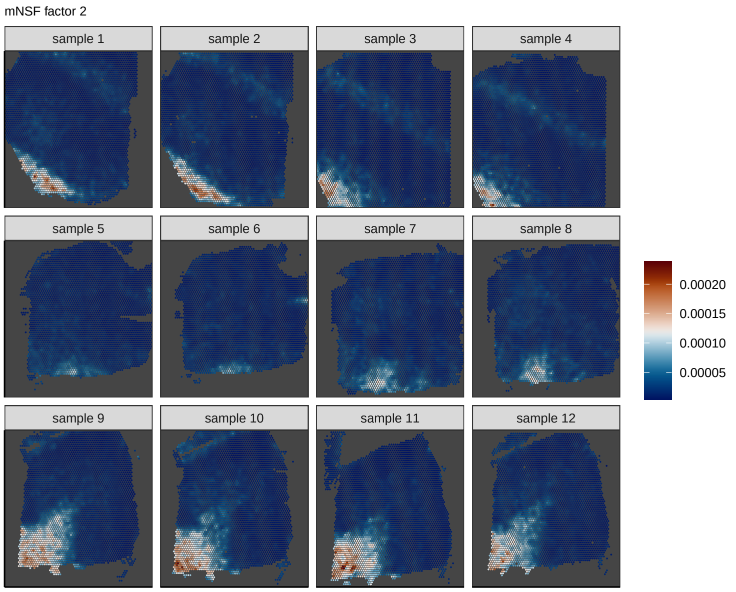

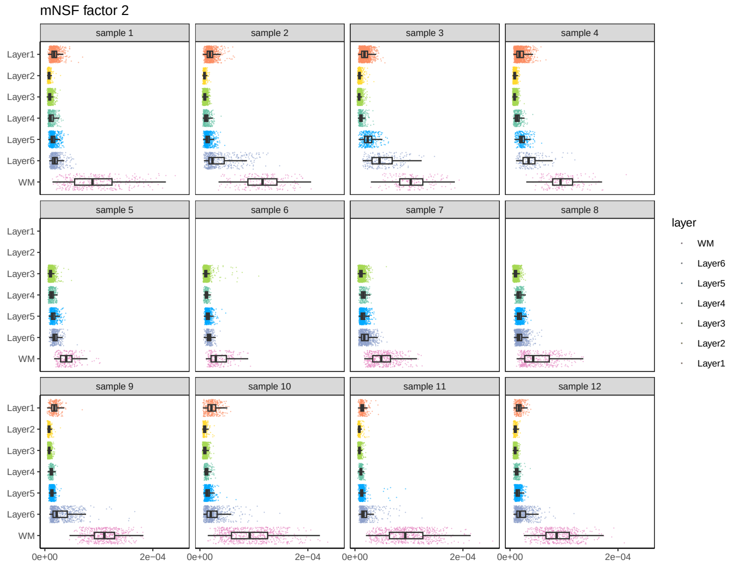

Fig S7. The value of each mNSF factor M2 for each of the 12 samples in DLPFC data

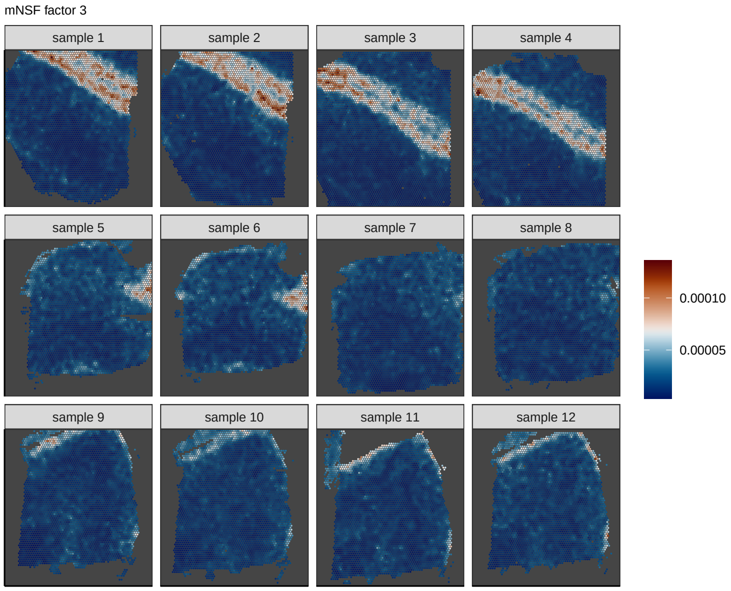

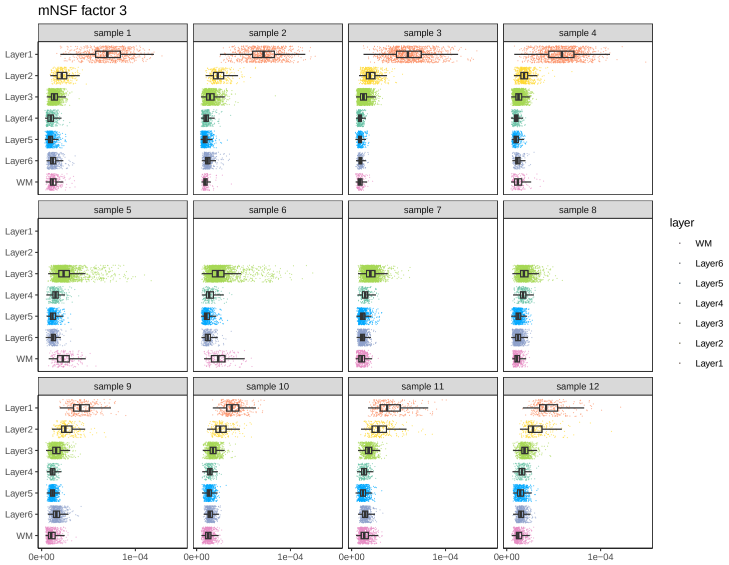

Fig S8. The value of each mNSF factor M3 for each of the 12 samples in DLPFC data

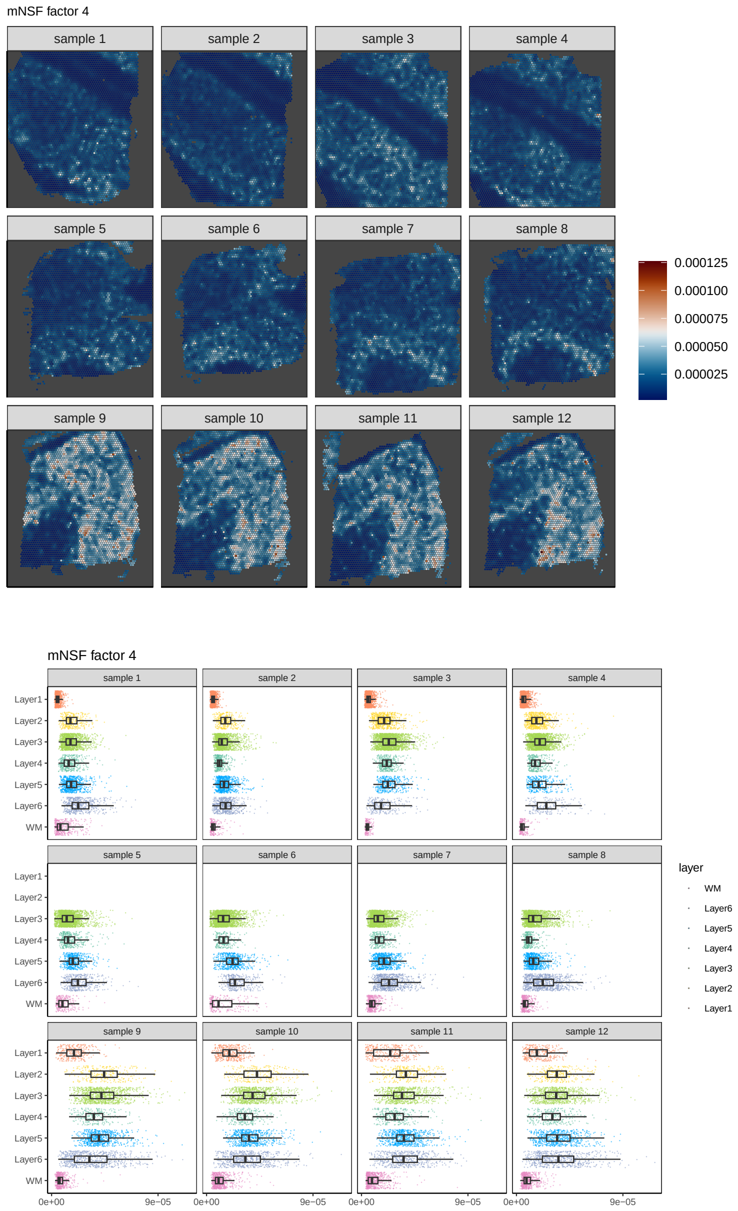

Fig S9. The value of each mNSF factor M4 for each of the 12 samples in DLPFC data

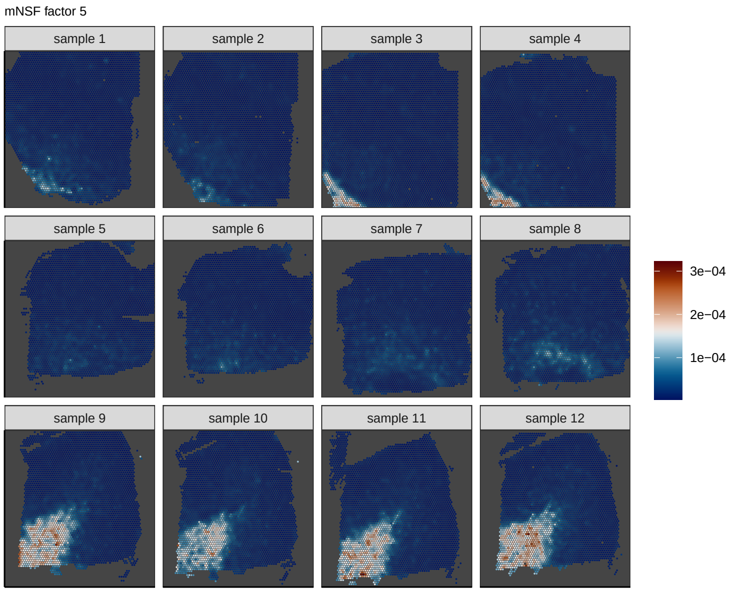

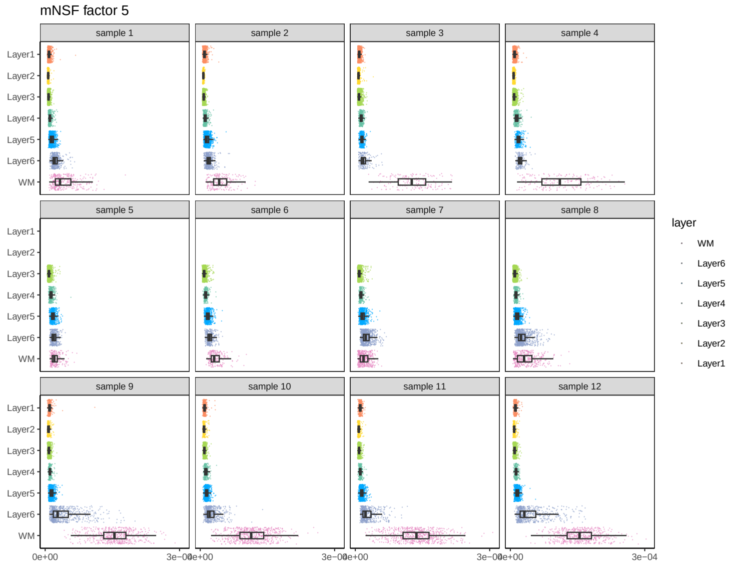

Fig S10. The value of each mNSF factor M5 for each of the 12 samples in DLPFC data

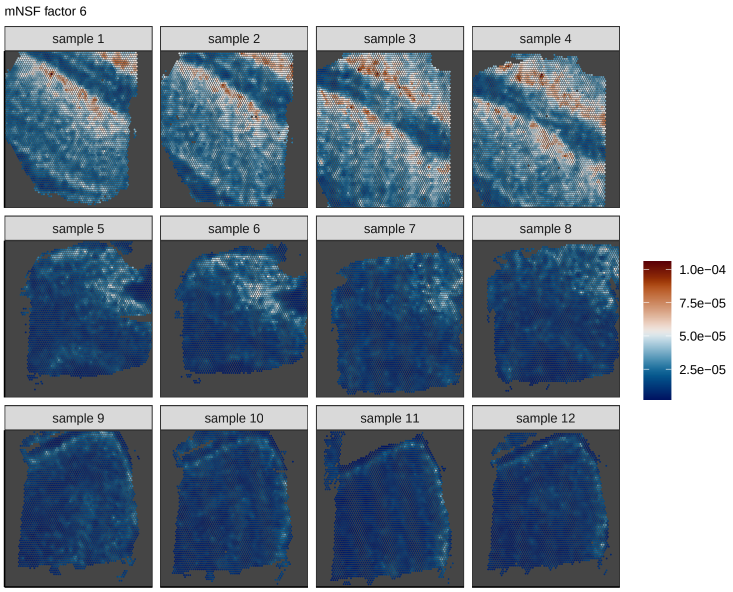

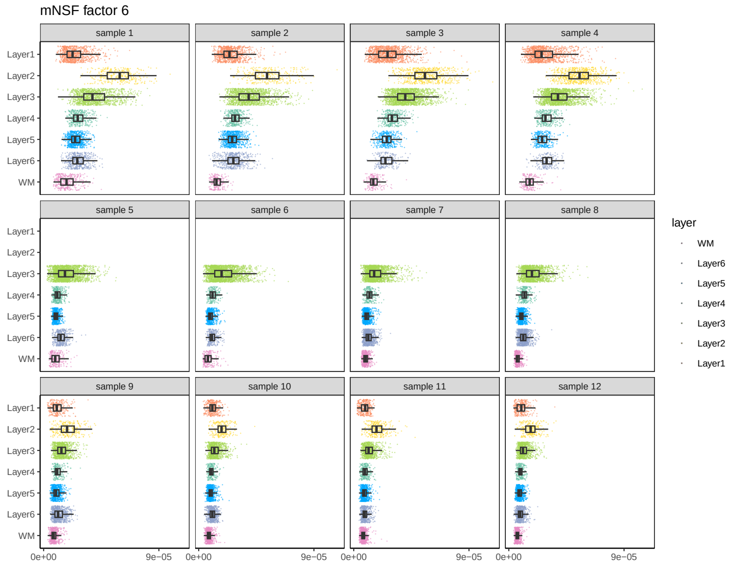

Fig S11. The value of each mNSF factor M6 for each of the 12 samples in DLPFC data

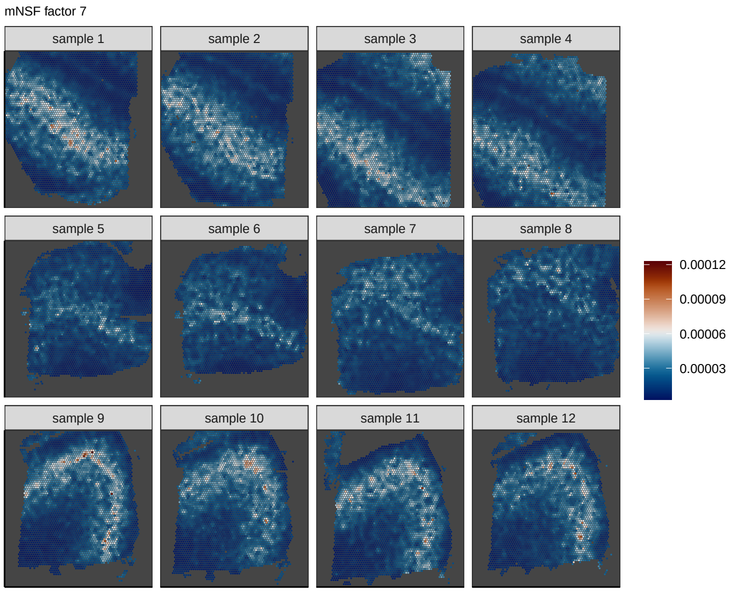

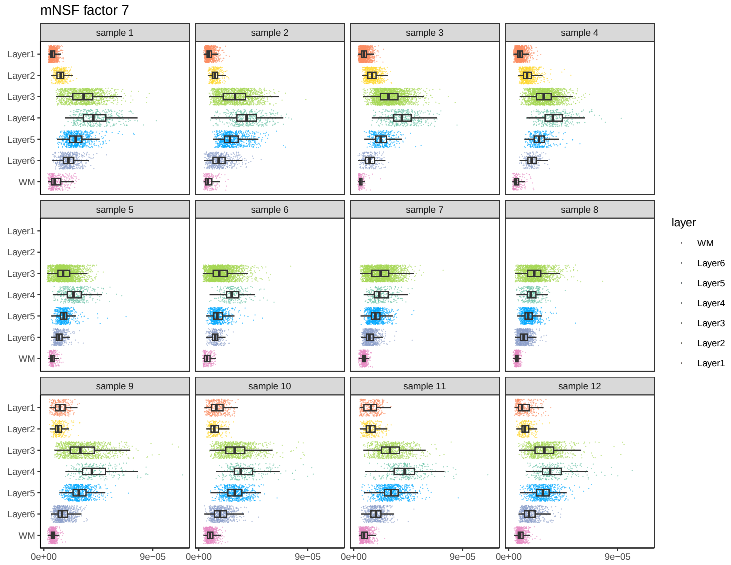

Fig S12. The value of each mNSF factor M7 for each of the 12 samples in DLPFC data

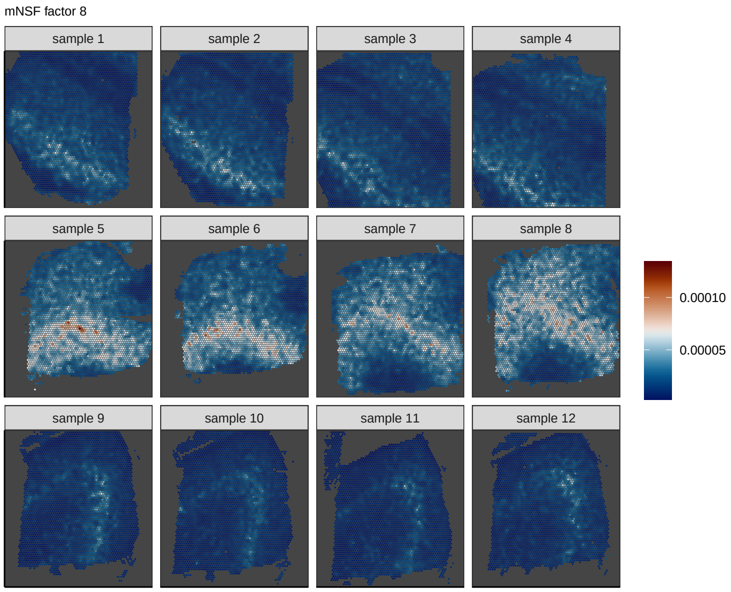

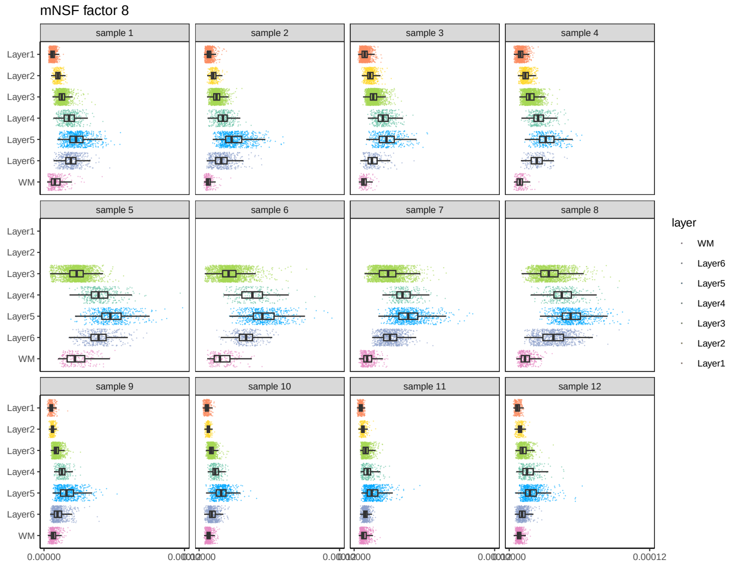

Fig S13. The value of each mNSF factor M8 for each of the 12 samples in DLPFC data

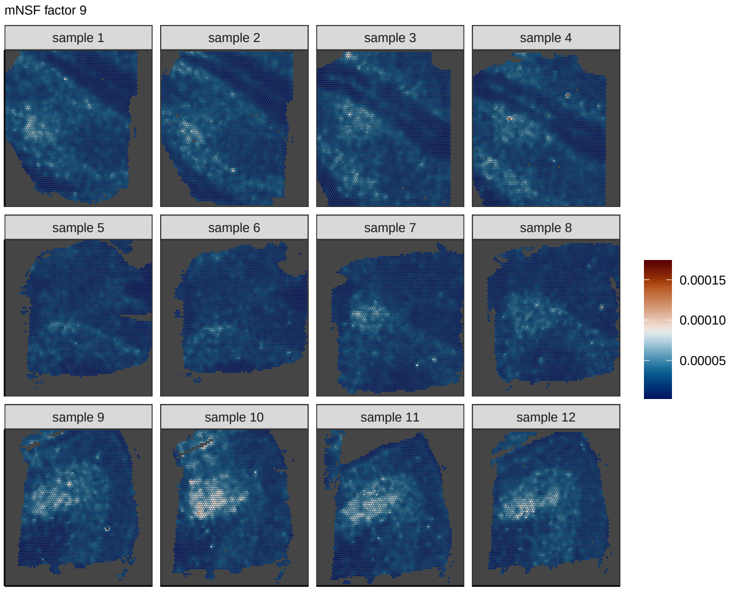

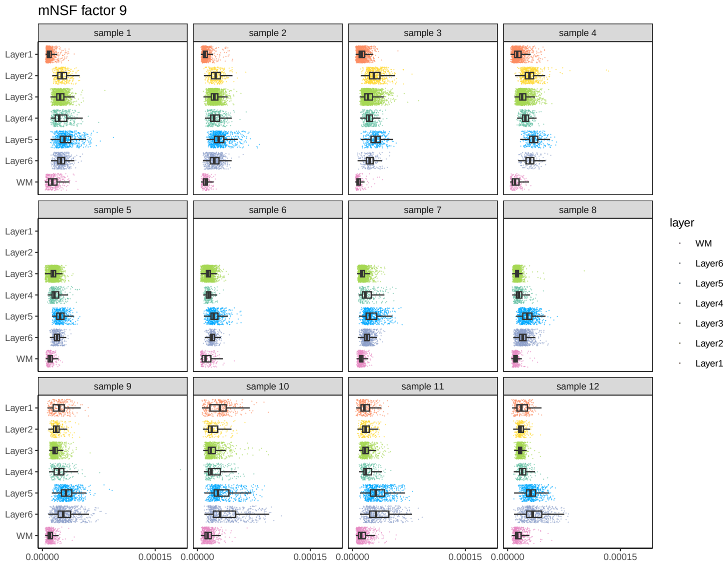

Fig S14. The value of each mNSF factor M9 for each of the 12 samples in DLPFC data

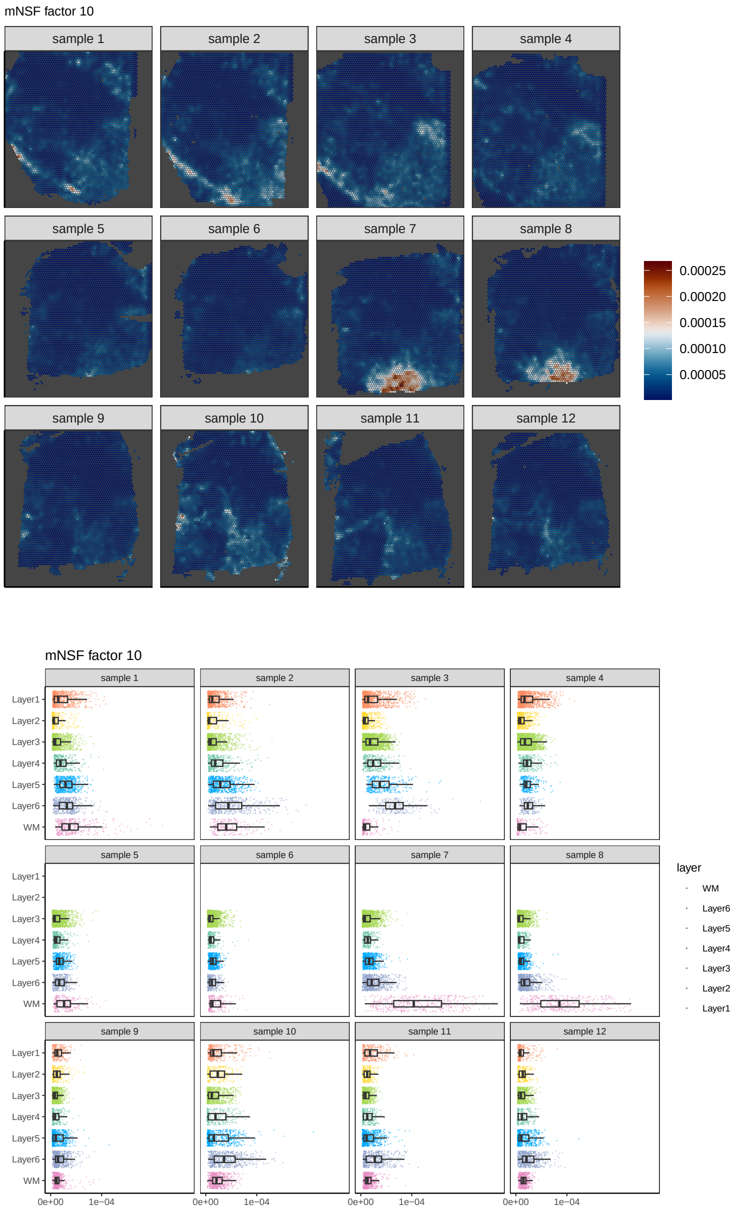

Fig S15. The value of each mNSF factor M10 for each of the 12 samples in DLPFC data

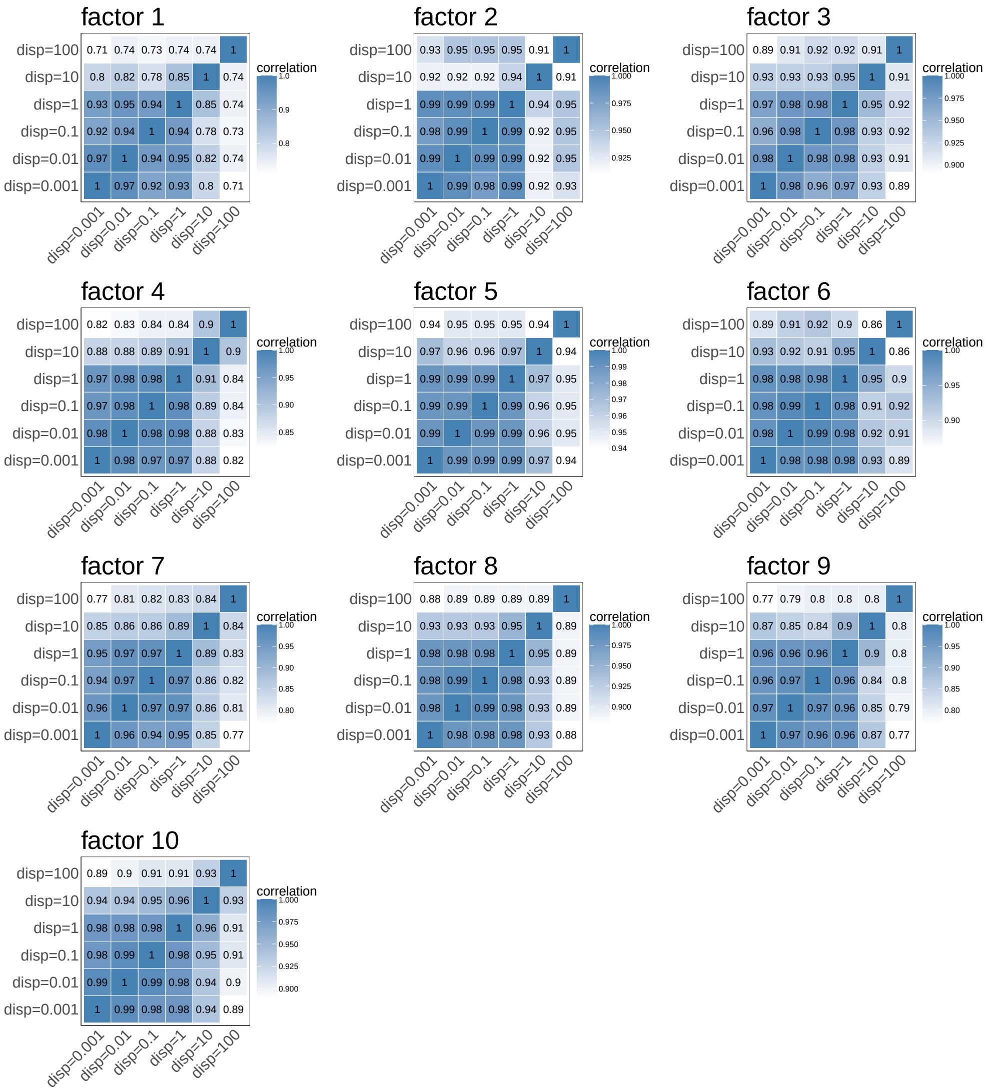

Fig S16. Sensitivity analysis of dispersion parameter on human DLPFC data. Correlation matrices showing the similarity between spatial factors obtained using different dispersion parameter values (ranging from 0.001 to 100) for each of the 10 factors identified in the DLPFC dataset. Each heatmap represents one factor, with colors indicating the correlation strength between factors obtained with different dispersion values. The high correlation values (> 0.8) observed across most parameter values for factors 1-10 indicate that mNSF results are generally robust to changes in the dispersion parameter within the range of 0.001 to 10, with some factors showing decreased correlation at dispersion=100.

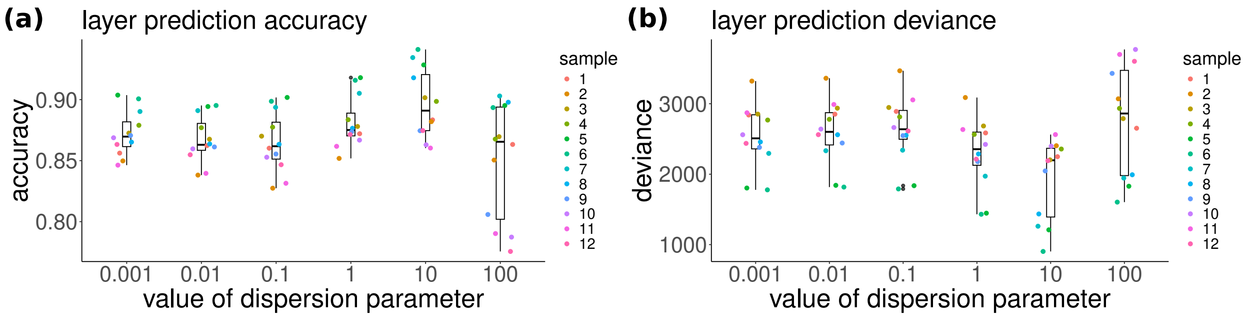

Fig S17. Performance of mNSF across dispersion parameter values on human DLPFC data. Analysis of how the dispersion parameter affects the association between mNSF factors and manually annotated cortical layers across 12 samples. We used a generalized linear model to predict layer and comapred to manual annotation on the training data. **(a)** Accuracy of our prediction. **(b)** Deviance measure for our prediction model (lower is better).

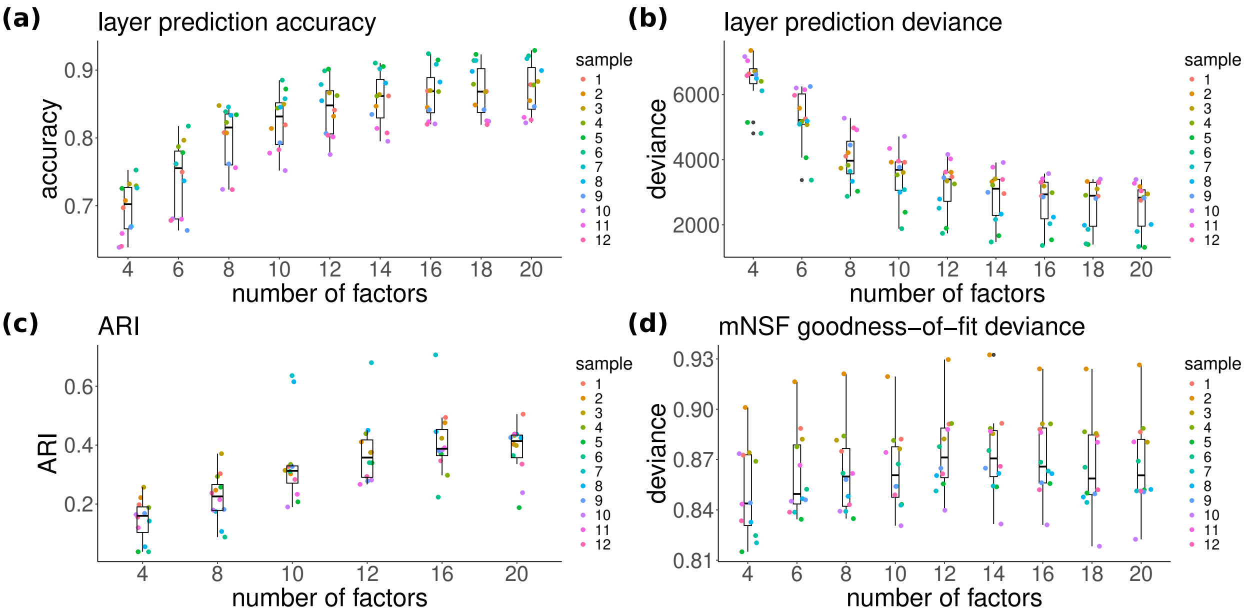

Fig S18. Evaluation of mNSF performance across different numbers of factors for DLPFC data. Systematic evaluation of mNSF performance using 4 to 20 factors across 12 samples. We used a generalized linear model to predict layer and comapred to manual annotation on the training data. **(a)** Accuracy of our model. **(b)** Deviance of our prediction model (lower is better). **(c)** Adjusted Rand Index (ARI): Quantifies the agreement between predicted spatial domains and known cortical layers. **(d)** mNSF goodness-of-fit deviance: measures the overall model fit using Poisson deviance between observed counts and predicted mean values.

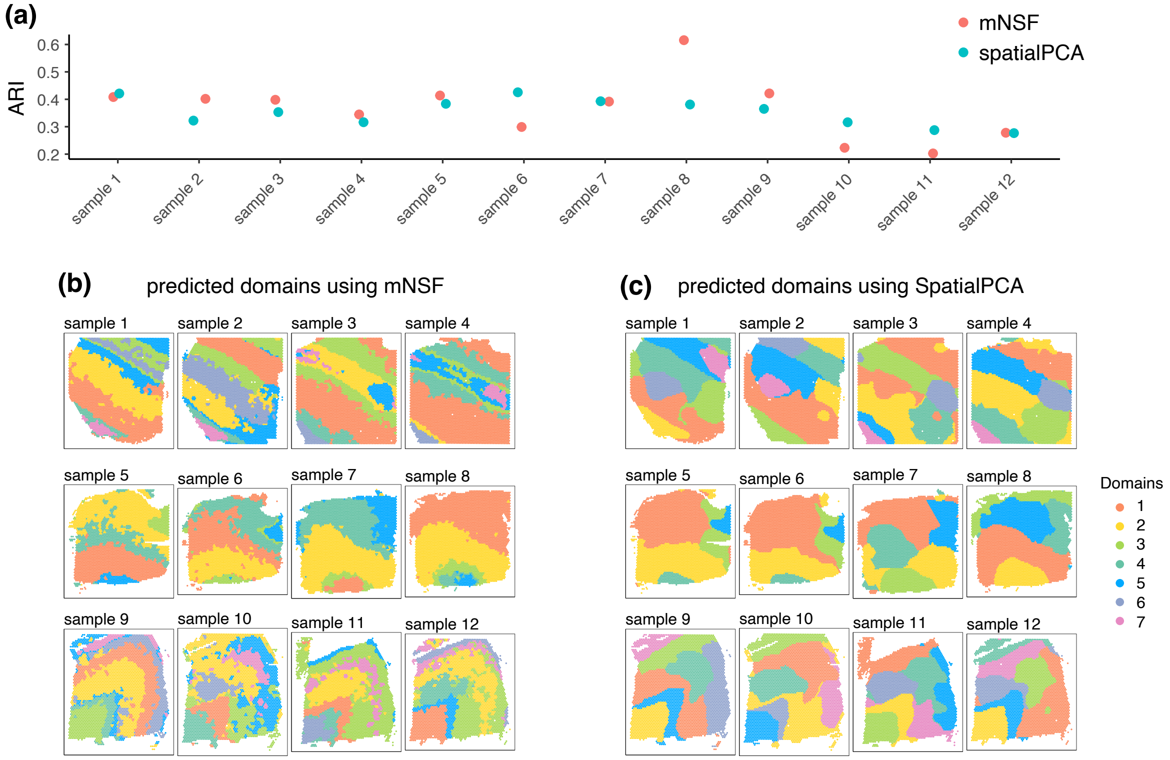

Fig S19. Comparison of domain identification performance between mNSF and SpatialPCA using the DLPFC dataset. (a) Adjusted Rand Index (ARI) scores comparing predicted domains to manually annotated layers across twelve samples. ARI values mostly ranged between 0.30 and 0.45, with neither method consistently outperforming the other across all samples. **(b)** Results of domain identification using mNSF, showing the predicted spatial domains for different samples. The colors represent distinct predicted domains within each sample. **(c)** Results of domain identification using SpatialPCA, showing comparable domain predictions for the same samples. The color scheme used is consistent within each method to facilitate comparison of domain structures.

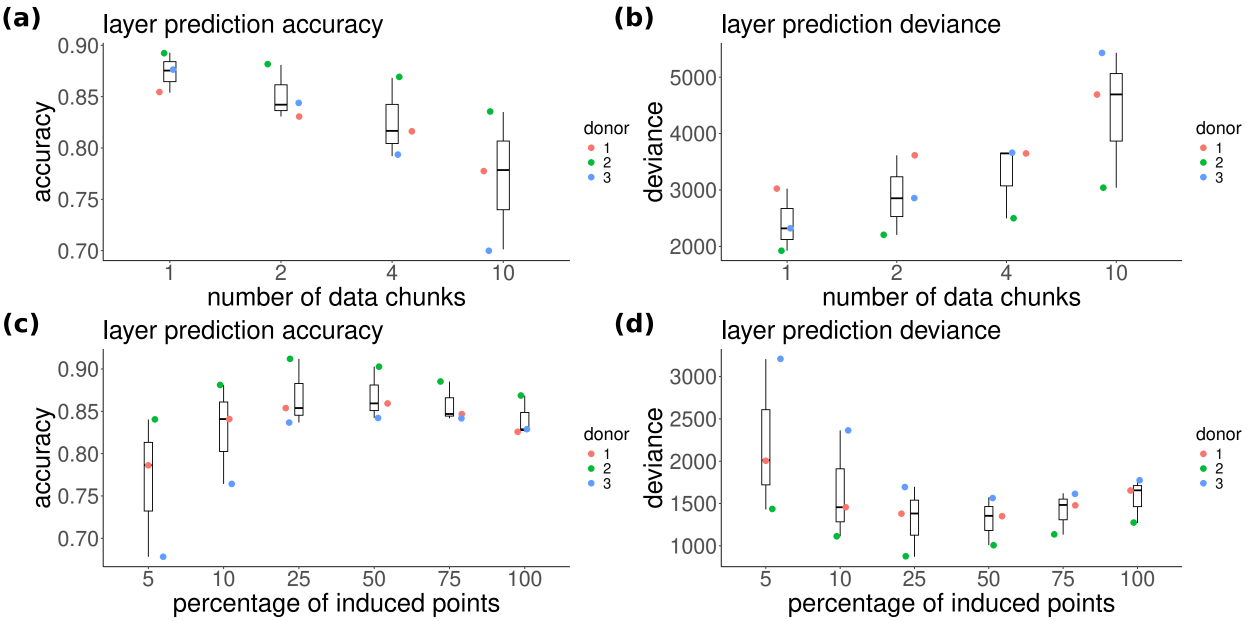

Fig S20. Performance evaluation of mNSF with varying induced points and data chunks on DLPFC data. Analysis of how induced points percentage and data chunking affect the association between mNSF factors and manually annotated cortical layers across three donors. **(a-b)** Layer prediction accuracy and deviance vs percentage of induced points. Higher accuracy and lower deviance indicate better performance. **(c-d)** Layer prediction accuracy and deviance vs number of data chunks.

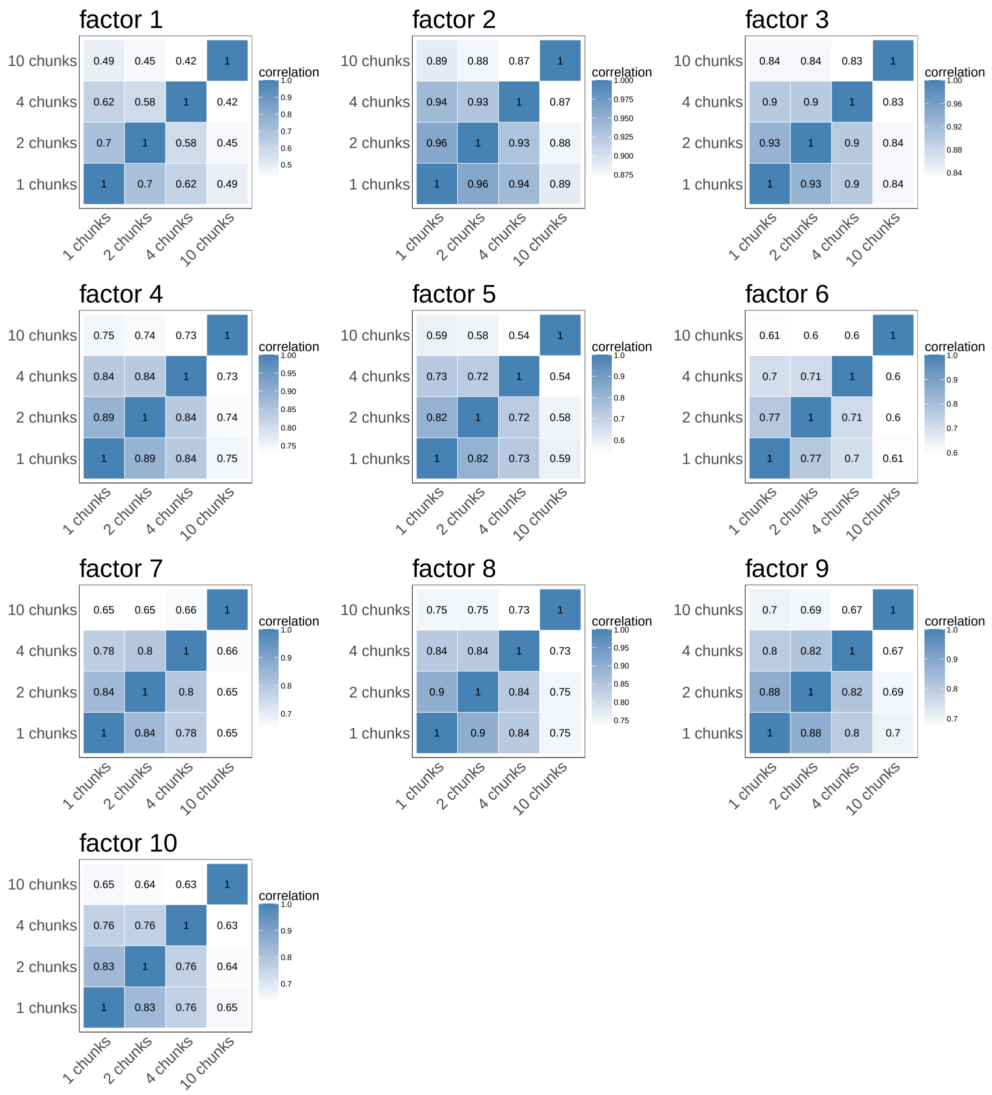

Fig S21. Impact of data chunking on factor correlation in DLPFC data. Correlation matrices showing the similarity between spatial factors obtained using different numbers of data chunks (1, 2, 4, and 10 chunks) for the 10 factors identified in the DLPFC dataset. Each heatmap represents one factor, with colors indicating the correlation strength between factors obtained with different chunk numbers. Lower correlations at higher chunk numbers suggest some loss of factor consistency with increased chunking.

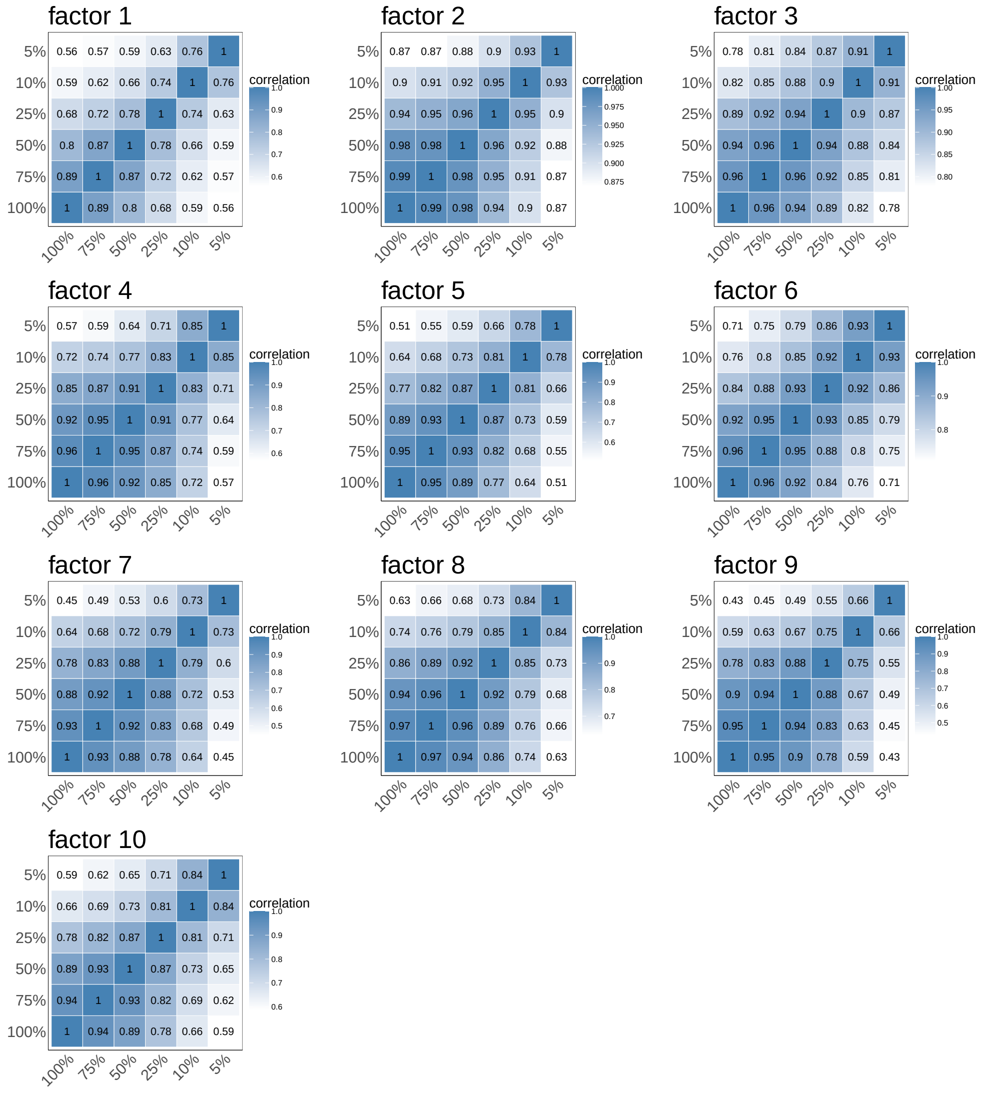

Fig S22. Effect of induced points percentage on factor stability in DLPFC data. Correlation matrices displaying the similarity between spatial factors obtained using different percentages of induced points (ranging from 5% to 100%) for factors 1-10. Each heatmap represents one factor, with correlation values indicated by color intensity. The high correlation values (generally > 0.8) between most percentage levels demonstrate that mNSF produces stable results across a broad range of induced point percentages, though some factors show decreased correlation at lower percentages.

2 Supplemental Tables

Factor	Gene symbol
M1	Tcf7l2, Bc1, Acta2, Slc17a6, Dcn, Trh, Tnnt1, Cabp7, Atp2a3, Ccn2
M2	Ttr, Enpp2, Ecrg4
M3	
M4	2010300C02Rik, Arpp21, Ppp3ca, Cx3cl1, Lamp5, Rgs4, Chst1, Pdp1, Ndrg4, Kcnip2
M5	Hba-a1, Hbb-bs, Hba-a2, Hbb-bt, Alas2
M6	mt-Co1, mt-Nd5, mt-Co2, mt-Atp8, mt-Nd2, mt-Nd4, mt-Atp6, mt-Nd4l, mt-Co3, mt-Nd3
M7	Lypd1, Ly6h, Pgrmc1, Hap1, Lmo3, Gap43, Ccn3, Crym, Atp2b4, Ahi1
M8	Vxn, Stx1a, Lingo1, Dkk3, Tbr1, Cck, Mef2c, Nrn1, 1110008P14Rik, Slc30a3
M9	Pcp2, Car8, Cbln1, Rgs8, Calb1, Itpr1, Cbln3, Gng13, Zic1, Inpp5a
M10	Penk, Gpr88, Ppp1r1b, Pde10a, Tac1, Pde1b, Rgs9, Adcy5, Scn4b, Rasd2
M11	Scg2, Nap1l5, Resp18, Tuba1b, Gnas
M12	Prkcd, Adarb1, Nefm, Cplx1, Slc24a2, Rasgrp1, Uchl1, Thy1, Atp1a3
M13	Tmsb10, Fxyd6, Rpl37, Rplp1, Rpl13, Rpl9, Rps19, Rps27, Rpl37a, Clu
M14	Ptgds, Mgp, Igf2, Myoc, Nnat, Igfbp2
M15	Plp1, Mobp, Mbp, Trf, Mag, Mal, Cldn11, Cryab, Cnp, Mog
M16	Fabp7, S100a5, Slc6a11, Ptn, Apoe, Nrsn1, Aqp4, Vtn, Sparcl1, Pla2g7
M17	Cnih2, Ddn, Ptk2b, Nptxr, Ncdn, Nsmf, Nell2, Mmd, Thra, Selenow
M18	Sst, Npy, Gad1, Gad2, Slc32a1, Zwint, Pcsk1n, Cox8a, Snrpn, Cox6c
M19	Gng4, Synpr, Gpsm1, Pcbp3, Meis2, Cpne4, Ptpro, Tshz1, Pbx3, Pcp4l1
M20	Gm42418, Lars2, Nefh, Vamp1, Spp1, Malat1, Nefl, Nat8l

Table S1: Genes mostly associated with each factor in mouse sagittal section data |

Factor	Gene symbol
M1	COX1, COX2, COX3, ND4, ATP6, ND2, ND3, CYTB, ND1, ND5
M2	KRT8, KRT18, S100A11, MOG, MOBP, HSPA2, BCAS1, IGFBP5, MBP, PAQR6
M3	FABP4, SAA1, AQP4, SNORC, CXCL14, VIM, SPARC, GJA1, GFAP, MT2A
M4	PPP3CA, DIRAS2, AK5, APP, THY1, PRKCB, CHN1, YWHAG, RTN4, PRNP
M5	PLP1, TF, CNP, CARNS1, HBA2, HBB, CLDND1, CLDN11, ENPP2, PPP1R14A
M6	HPCAL1
M7	NEFM, NEFL, SNCG, LGALS1, GAP43
M8	PCP4, SNCA, TUBB2A, TMSB10, SYT1, STMN2, STMN1, UCHL1, FABP3, TTC9B
M9	COX6C, SST, NPY
M10	SCGB2A2, SCGB1D2, TFF1, IGKC, IGHG3, AZGP1, IGHG4, TFF3, MUC1, IGLC2

Table S2: Genes associated with each factor in the DLPFC data |

Dataset	Number of Factors	Initial Peak Memory (GB)	Final Peak Memory (GB)	Runtime (seconds)
Simulation	4	0.1	1.6	215
Mouse sagittal section	20	1.1	15.6	1,193
DLPFC	10	1.9	17.6	1,804
Cerebellum	10	0.9	8.7	1,264

Table S3: Memory consumption and runtime analysis for different spatial transcriptomics datasets. The table shows the memory requirements at different stages of the analysis pipeline and the total computational time required for each dataset.
